# Supplementary material for: Transgenerational inheritance of ethanol preference is caused by maternal NPF repression
Source: eLife. 2019 Jul 9;8:e45391. doi: 10.7554/eLife.45391 (PMC6615861; doi:10.7554/eLife.45391)
Supplement: Supplementary file 1. [file elife-45391-supp1.docx]

Supplementary Materials for

Transgeneratonal inheritance of ethanol preference is caused by maternal NPF repression.

Julianna Bozler^1^, Balint Z Kacsoh^1^, Giovanni Bosco^1^*

Correspondence to: Giovanni.Bosco@Dartmouth.edu

**Supplementary file 1**. Statistical tests and p-values relating to main text figures.

| **Figure** | **Comparison groups** | **p-value** | **statistical test** |
| --- | --- | --- | --- |
| 1B | F0 (Exposed vs unexposed) | 1.08E-05 | Mann-Whitney Rank Sum |
| 1B | F1 (Exposed vs unexposed) | 8.64E-07 | Mann-Whitney Rank Sum |
| 1B | F2 (Exposed vs unexposed) | 2.58E-08 | Mann-Whitney Rank Sum |
| 1B | F3 (Exposed vs unexposed) | 1.29E-08 | Mann-Whitney Rank Sum |
| 1B | F4 (Exposed vs unexposed) | 4.13E-06 | Mann-Whitney Rank Sum |
| 1B | F5 (Exposed vs unexposed) | 0.00833 | Mann-Whitney Rank Sum |
| 1B | F6 (Exposed vs unexposed) | 0.6063 | Mann-Whitney Rank Sum |
| 1C | CS-during (Exposed vs unexposed) | 0.0001817 | Mann-Whitney Rank Sum |
| 1C | Orb2-during (Exposed vs unexposed) | 0.000278 | Mann-Whitney Rank Sum |
| 1C | CS-post (Exposed vs unexposed) | 1.08E-05 | Mann-Whitney Rank Sum |
| 1C | Orb2-post (Exposed vs unexposed) | 0.02065 | Mann-Whitney Rank Sum |
| 2A | F0 (Exposed vs unexposed) | 0.0001593 | Mann-Whitney Rank Sum |
| 2A | F1 (Exposed vs unexposed) | 0.3144 | Mann-Whitney Rank Sum |
| 2B | High protein vs low protein | 0.0001079 | Mann-Whitney Rank Sum |
| 2C | Drice[RNAi] (Exposed vs unexposed) | 0.5787 | Mann-Whitney Rank Sum |
| 2C | Dcp-1[RNAi] (Exposed vs unexposed) | 0.05889 | Mann-Whitney Rank Sum |
| 2D | High protein vs low protein | 0.933864 | Mann-Whitney Rank Sum |
| 3A | NPF OE (Exposed vs unexposed) | 0.5787 | Mann-Whitney Rank Sum |
| 3A | NPF KD vs NPF-Gal4 | 1.08E-05 | Mann-Whitney Rank Sum |
| 3B | NPF OE (Exposed vs unexposed) | 0.1758 | Mann-Whitney Rank Sum |
| 3B | NPF KD vs NPF-Gal4 | 1.76E-05 | Mann-Whitney Rank Sum |
| 3C | Elav-Gal4 (Exposed vs unexposed) | 0.0001697 | Mann-Whitney Rank Sum |
| 3C | NPFR[RNAi] (Exposed vs unexposed) | 3.07E-06 | Mann-Whitney Rank Sum |
| 3C | NPFR KD (Exposed vs unexposed) | 0.0007069 | Mann-Whitney Rank Sum |
| 3C | NPFR KD vs NPFR[RNAi] unexposed | 0.0001503 | Mann-Whitney Rank Sum |
| 3C | NPFR KD vs Elav-Gal4 unexpoed | 0.0006232 | Mann-Whitney Rank Sum |
| 3D | NPF OE (Exposed vs unexposed) | 0.4359 | Mann-Whitney Rank Sum |
| 3D | NPF KD (Exposed vs unexposed) | 1.08E-05 | Mann-Whitney Rank Sum |
| 3D | NPF-Gal4 (Exposed vs unexposed) | 1.08E-05 | Mann-Whitney Rank Sum |
| 3D | NPF KD vs NPF-Gal4 unexposed | 1.08E-05 | Mann-Whitney Rank Sum |
| 3E | Elav-Gal4 (Exposed vs unexposed) | 0.0001817 | Mann-Whitney Rank Sum |
| 3E | NPFR[RNAi] (Exposed vs unexposed) | 0.0001817 | Mann-Whitney Rank Sum |
| 3E | NPFR KD (Exposed vs unexposed) | 0.0001817 | Mann-Whitney Rank Sum |
| 3E | NPFR KD vs NPFR[RNAi] unexposed | 1.08E-05 | Mann-Whitney Rank Sum |
| 3E | NPFR KD vs Elav-Gal4 unexpoed | 0.0001817 | Mann-Whitney Rank Sum |
| 3F | NPF OE (Exposed vs unexposed) | 0.7333 | Mann-Whitney Rank Sum |
| 4B | F0 (Exposed vs unexposed) | 0.009027 | Mann-Whitney Rank Sum |
| 4B | F1 (Exposed vs unexposed) | 0.0004949 | Mann-Whitney Rank Sum |
| 4B | F2 (Exposed vs unexposed) | 0.002572 | Mann-Whitney Rank Sum |
| 4C | F0 (Exposed vs unexposed) | 3.09E-11 | Mann-Whitney Rank Sum |
| 4C | F1 (Exposed vs unexposed) | 0.3972 | Mann-Whitney Rank Sum |
| 4C | F2 (Exposed vs unexposed) | 0.6378 | Mann-Whitney Rank Sum |
| 5A | maternal (Exposed vs unexposed) | 0.000011 | Mann-Whitney Rank Sum |
| 5A | paternal (Exposed vs unexposed) | 0.1904 | Mann-Whitney Rank Sum |
| 5B | Blind mothers (Exposed vs unexposed) | 0.3154 | Mann-Whitney Rank Sum |
| 5B | Blind fathers (Exposed vs unexposed) | 0.0002712 | Mann-Whitney Rank Sum |
| 5D | Chr-II Maternal C(2)EN b[1] pr[1] (Exposed vs unexposed) | 0.0001796 | Mann-Whitney Rank Sum |
| 5D | Chr-II Paternal C(2)EN b[1] pr[1] (Exposed vs unexposed) | 0.0002695 | Mann-Whitney Rank Sum |
| 5D | Chr-II Maternal C(2)EN bw[1] sp[1] (Exposed vs unexposed) | 0.0004456 | Mann-Whitney Rank Sum |
| 5D | Chr-II Paternal C(2)EN bw[1] sp[1] (Exposed vs unexposed) | 0.0002695 | Mann-Whitney Rank Sum |
| 5D | Chr-III Maternal C(3)EN Diap1[1] sp[1] (Exposed vs unexposed) | 0.0006306 | Mann-Whitney Rank Sum |
| 5D | Chr-III Paternal C(3)EN Diap1[1] sp[1] (Exposed vs unexposed) | 0.7308 | Mann-Whitney Rank Sum |
| 5D | Chr-III Maternal C(3)EN st[1] cu[1] e[s] (Exposed vs unexposed) | 0.0006258 | Mann-Whitney Rank Sum |
| 5D | Chr-III Paternal C(3)EN st[1] cu[1] e[s](Exposed vs unexposed) | 0.8857 | Mann-Whitney Rank Sum |
| 5F | Maternal Df(3)ED10642 (Exposed vs unexposed) | 0.8796 | Mann-Whitney Rank Sum |
| 5F | Maternal ED10642-balancer (Exposed vs unexposed) | 0.0001766 | Mann-Whitney Rank Sum |
| 5F | Maternal Df(3)BCS472 (Exposed vs unexposed) | 0.7569 | Mann-Whitney Rank Sum |
| 5F | Maternal BCS472-balancer (Exposed vs unexposed) | 0.000278 | Mann-Whitney Rank Sum |
| 5F | Maternal Df(3)BCS510 (Exposed vs unexposed) | 0.002141 | Mann-Whitney Rank Sum |
| 5F | Maternal BCS510-balancer (Exposed vs unexposed) | 0.002141 | Mann-Whitney Rank Sum |
| 5F | Paternal Df(3)ED10642 (Exposed vs unexposed) | 0.0001756 | Mann-Whitney Rank Sum |
| 5F | Paternal ED10642-balancer (Exposed vs unexposed) | 0.0001796 | Mann-Whitney Rank Sum |
| 5F | Paternal Df(3)BCS472 (Exposed vs unexposed) | 0.0004426 | Mann-Whitney Rank Sum |
| 5F | Paternal BCS472-balancer (Exposed vs unexposed) | 0.0002451 | Mann-Whitney Rank Sum |
| 5F | Paternall Df(3)BCS510 (Exposed vs unexposed) | 0.000278 | Mann-Whitney Rank Sum |
| 5F | Paternal BCS510-balancer (Exposed vs unexposed) | 0.0001817 | Mann-Whitney Rank Sum |
| S1A | F1 During wasp exposure(Exposed vs unexposed) | 0.0001806 | Mann-Whitney Rank Sum |
| S1A | F1 Post wasp exposure(Exposed vs unexposed) | 0.2113 | Mann-Whitney Rank Sum |
| S1B | F1 During wasp exposure: Methanol (Exposed vs unexposed) | 0.0001817 | Mann-Whitney Rank Sum |
| S1B | F1 During wasp exposure: RU486 (Exposed vs unexposed) | 0.0001786 | Mann-Whitney Rank Sum |
| S1B | F1 Post wasp exposure: Methanol (Exposed vs unexposed) | 0.0003955 | Mann-Whitney Rank Sum |
| S1B | F1 Post wasp exposure: RU486 (Exposed vs unexposed) | 0.791 | Mann-Whitney Rank Sum |
| S2D | Brood 1 (Exposed vs unexposed) | 2.49E-10 | Mann-Whitney Rank Sum |
| S2D | Brood 2 (Exposed vs unexposed) | 0.6305 | Mann-Whitney Rank Sum |
| S2E | Exposed (1 gen) vs unexposed | 1.08E-05 | Mann-Whitney Rank Sum |
| S2E | Exposed (2 gen) vs unexposed | 1.08E-05 | Mann-Whitney Rank Sum |
| S2E | Exposed (1 gen) vs exposed (2 gen) | 1.08E-05 | Mann-Whitney Rank Sum |
| S2F | Exposed (1 gen) vs unexposed | 1.82E-04 | Mann-Whitney Rank Sum |
| S2F | Exposed F8 (2 gen) vs unexposed | 1.08E-05 | Mann-Whitney Rank Sum |
| S2F | Exposed (1 gen) vs exposed F8 (2 gen) | 0.472 | Mann-Whitney Rank Sum |
| S3A | Exposed vs unexposed | 1.08E-05 | Mann-Whitney Rank Sum |
| S3B | Exposed vs unexposed | 0.000181 | Mann-Whitney Rank Sum |
| S5 | F0 (Exposed vs unexposed) | 0.3429 | students t-test |
| S5 | F1 (Exposed vs unexposed) | 0.3429 | students t-test |
| S6 | UAS-NPF[RNAi] (Exposed vs unexposed) | 0.000278 | Mann-Whitney Rank Sum |
| S6 | UAS-NPF (Exposed vs unexposed) | 1.08E-05 | Mann-Whitney Rank Sum |
