# Supplementary material for: Transgenerational inheritance of ethanol preference is caused by maternal NPF repression
Source: eLife. 2019 Jul 9;8:e45391. doi: 10.7554/eLife.45391 (PMC6615861; doi:10.7554/eLife.45391)
Supplement: Supplementary file 2. — Key experiments were replicated using the additional wild-type strain OreR. ‘Corresponding Figure’ indicates the experiment that was replicated: A listing of Figure 1B therefore indicates that the experimental conditions for Figure 1B were duplicated using OreR flies. [file elife-45391-supp2.docx]

**Supplementary file 2**. Oregon R experimental data. Key experiments were replicated using the additional wild-type strain OreR. “Corresponding Figure” indicates the experiment that was replicated: A listing of Fig1B therefore indicates that the experimental conditions for Figure 1B were duplicated using OreR flies.

|  |  | **Day 1** |  |  | **Day 2** |  |  |
| --- | --- | --- | --- | --- | --- | --- | --- |
| **Corresponding Figure**  **for Duplicate**  **Experiment** | **Description** | **Mean**  **(experimental)** | **Mean**  **(control)** | **p-value** | **Mean**  **(experimental)** | **Mean**  **(control)** | **p-value** |
| 1B | F0 | 0.913 | 0.288 | 1.08E-05 | 0.927 | 0.276 | 1.08E-05 |
| 1B | F1 | 0.704 | 0.303 | 1.08E-05 | 0.695 | 0.276 | 1.81E-04 |
| 1B | F2 | 0.679 | 0.286 | 1.08E-05 | 0.691 | 0.271 | 1.82E-04 |
| 1B | F3 | 0.675 | 0.25 | 1.08E-05 | 0.647 | 0.266 | 1.82E-04 |
| 1B | F4 | 0.556 | 0.211 | 2.44E-04 | 0.534 | 0.221 | 1.08E-05 |
| 1B | F5 | 0.366 | 0.277 | 0.07526 | 0.41 | 0.221 | 0.0001299 |
| 1B | F6 | 0.273 | 0.263 | 0.7394 | 0.224 | 0.238 | 0.6305 |
| Not shown in figure | F7 | 0.202 | 0.208 | 0.4359 | 0.211 | 0.193 | 0.3429 |
| 2A | F0 apoptosis (Exposed vs unexposed) | 0.705 | 1.70E-02 | 0.0001442 |  |  |  |
| 2A | F1 apoptosis (Exposed vs unexposed) | 0.017 | 3.10E-02 | 0.2931 |  |  |  |
| 5A | Paternal | 0.36 | 0.45 | 0.1655 | 0.34 | 0.35 | 0.8534 |
| S2D | Brood 2 | 0.23 | 0.27 | 0.705 | 0.24 | 0.27 | 0.6842 |
| S2E | Exposed (1 gen) vs unexposed | 0.744 | 0.213 | 1.81E-04 | 0.723 | 0.186 | 1.82E-04 |
| S2E | Exposed (2 gen) vs unexposed | 0.91 | - | 1.08E-05 | 0.924 | - | 1.82E-04 |
| S2E | Exposed (1 gen) vs exposed (2 gen) | - | - | 1.81E-04 | - | - | 1.81E-04 |
| S2F | Exposed (1 gen) vs unexposed | 0.79 | 0.258 | 1.82E-04 | 0.81 | 0.223 | 1.82E-04 |
| S2F | Exposed F8 (2 gen) vs unexposed | 0.76 | - | 1.82E-04 | 0.792 | - | 1.80E-04 |
| S2F | Exposed (1 gen) vs exposed F8 (2 gen) | - | - | 0.1209 | - | - | 0.7048 |
