# Supplementary material for: Transgenerational inheritance of ethanol preference is caused by maternal NPF repression
Source: eLife. 2019 Jul 9;8:e45391. doi: 10.7554/eLife.45391 (PMC6615861; doi:10.7554/eLife.45391)
Supplement: Supplementary file 3. [file elife-45391-supp3.docx]

**Supplementary file 3**. Canton S day-2 data; mean(s) and p-value(s).

| **Corresponding**  **Figure** | **Description** | **Mean**  **(experimental)** | **Mean**  **(control)** | **p-value** |
| --- | --- | --- | --- | --- |
| 1B | F0 | 0.959 | 0.201 | 2.71E-04 |
| 1B | F1 | 0.8 | 0.369 | 3.38E-06 |
| 1B | F2 | 0.689 | 0.349 | 1.29E-08 |
| 1B | F3 | 0.595 | 0.258 | 6.12E-06 |
| 1B | F4 | 0.523 | 0.262 | 2.58E-08 |
| 1B | F5 | 0.431 | 0.267 | 0.01256 |
| 1B | F6 | 0.237 | 0.264 | 0.1577 |
| Not shown in figure | F7 | 0.158 | 0.163 | 0.7674 |
| 2C | Drice[RNAi] | 0.295 | 0.291 | 0.6774 |
| 2C | Dcp-1[RNAi] | 0.329 | 0.261 | 0.4359 |
| 2D | Low protein v high (control) | 0.163 | 0.173 | 0.575213 |
| 5A | maternal | 0.754 | 0.244 | 0.000182 |
| 5A | paternal | 0.425 | 0.29 | 0.001031 |
| S2D | Brood 1 | 0.81 | 0.335 | 3.12E-08 |
| S2D | Brood 2 | 0.33 | 0.273 | 0.1903 |
| S2E | Exposed (1 gen) v Unexposed | 0.769 | 0.164 | 1.08E-05 |
| S2E | Exposed (2 gen) v Exposed (1 gen) | 0.908 | - | 1.82E-04 |
| S2E | Exposed (2 gen) v Unexposed | - | - | 1.82E-04 |
| S2F | Exposed (1 gen) v Unexposed | 0.737 | 0.172 | 1.82E-04 |
| S2F | Exposed F8 (2 gen) v Exposed (1 gen) | 0.784 | - | 0.08873 |
| S2F | Exposed F8 (2 gen) v Unexposed | - | - | 1.82E-04 |
| S3A | paternal (F1) | 0.569 | 194 | 1.08E-05 |
| S3B | Two-week old F1 | 0.833 | 0.127 | 0.000022 |
