# Supplementary material for: Transgenerational inheritance of ethanol preference is caused by maternal NPF repression
Source: eLife. 2019 Jul 9;8:e45391. doi: 10.7554/eLife.45391 (PMC6615861; doi:10.7554/eLife.45391)
Supplement: Supplementary file 4. — The beta value (b) is approximately analogous to the natural log fold change of the transcript, and the q-value is the measure of significance. Transcript meeting the threshold criteria (q-value and beta) for any one generation was included in the table (corresponding to the head map of Figure 1—figure supplement 4). [file elife-45391-supp4.docx]

**Supplementary file 4**. RNA sequencing results from female fly heads across generations. The beta value (b) is approximately analogous to the natural log fold change of the transcript, and the q-value is the measure of significance. Transcript meeting the threshold criteria (q-value and beta) for any one generation was included in the table (corresponding to the head map of Fig S4).

| FBtr | Gene name | F0 (q-value) | F0 (b) | F1 (q-value) | F1 (b) | F2 (q-value) | F2 (b) |
| --- | --- | --- | --- | --- | --- | --- | --- |
| FBtr0070879 | vanin-like | 0.042794694 | 2.695203337 | 0.999860533 | 0.198884151 | 0.999798691 | -1.215423422 |
| FBtr0071534 | Cht8 | 0.006143547 | 2.99074439 | 0.999860533 | 0.14548922 | 0.999798691 | -1.32576327 |
| FBtr0071543 | Cht4 | 0.010179506 | 3.763386856 | 0.999860533 | 0.049037368 | 0.999798691 | -1.52411489 |
| FBtr0072121 | CG3906 | 0.000755348 | 3.097285784 | 0.999860533 | 0.132973058 | 0.999798691 | -1.205036739 |
| FBtr0072879 | CG13806 | 0.000427043 | 3.340054966 | 0.999860533 | -0.274578725 | 0.999798691 | -1.2348752 |
| FBtr0072938 | CG1246 | 0.001327451 | 4.323284994 | 0.999860533 | 1.496645518 | 0.999798691 | -0.011164927 |
| FBtr0073059 | Drsl2 | 0.006860599 | 2.791972648 | 0.999860533 | -0.086738812 | 0.999798691 | -0.532385558 |
| FBtr0073073 | Drs | 0.049913638 | 1.424942618 | 0.999860533 | 0.234538801 | 0.999798691 | 0.057697516 |
| FBtr0074375 | CG15865 | 9.56E-09 | 2.330606904 | 0.999860533 | 0.040608772 | 0.999798691 | -0.846980654 |
| FBtr0074393 | CG5162 | 0.999961787 | 2.490317828 | NA | NA | 0.018783539 | 5.381305094 |
| FBtr0074466 | CG32557 | 0.01108192 | 4.061506316 | 0.999860533 | -0.004714052 | 0.999798691 | -1.504733781 |
| FBtr0074626 | CG15043 | 2.25E-06 | 4.756372803 | 0.999860533 | -0.639492849 | 0.999798691 | -1.581079437 |
| FBtr0075048 | CG3819 | 0.049183959 | 4.488850505 | 0.999860533 | -1.059384579 | 0.999798691 | -1.509328776 |
| FBtr0075069 | CG6839 | 0.00017266 | 3.983281993 | 0.999860533 | -0.193649236 | 0.999798691 | -1.571518506 |
| FBtr0075157 | CG5506 | 0.022720543 | 3.759914041 | 0.999860533 | 0.000937011 | 0.999798691 | -1.233159619 |
| FBtr0075908 | ara | 0.029084942 | 4.631010546 | 0.999860533 | -1.603859525 | 0.999798691 | -3.018228464 |
| FBtr0076044 | CG14125 | 0.002352177 | 5.47281471 | 0.999860533 | -0.068130761 | 0.999798691 | -1.877924062 |
| FBtr0076063 | Muc68E | 8.69E-07 | 3.848174764 | 0.999860533 | -0.338540181 | 0.999798691 | -1.187233981 |
| FBtr0076119 | Muc68D | 0.000716285 | 3.009816876 | 0.999860533 | 0.261433904 | 0.999798691 | -1.286920059 |
| FBtr0076263 | simj | NA | NA | 0.999860533 | 3.317436292 | 0.012996463 | 5.803771619 |
| FBtr0076316 | CG14160 | 0.038017334 | 2.502603348 | 0.999860533 | 0.048202942 | 0.999798691 | -1.30267021 |
| FBtr0077038 | yip7 | 0.024246802 | 4.10158033 | 0.999860533 | -0.493209078 | 0.999798691 | -1.840600344 |
| FBtr0077041 | Jon65Aiii | 0.009550923 | 3.267952398 | 0.999860533 | -0.38746908 | 0.999798691 | -1.582082054 |
| FBtr0077220 | lcs | 6.05E-09 | -1.490020237 | 0.999860533 | 0.446269639 | 0.999798691 | 0.757663199 |
| FBtr0077465 | ed | NA | NA | 0.013747666 | 5.462236135 | 0.70253039 | 4.102767521 |
| FBtr0078908 | CG14645 | 0.000861022 | 2.982720707 | 0.999860533 | 0.163951243 | 0.999798691 | -1.081638609 |
| FBtr0078953 | Skp2 | 7.62E-07 | 1.383516598 | 0.999860533 | 0.095441181 | 0.999798691 | -0.627969812 |
| FBtr0079136 | CG11029 | 1.23E-07 | 1.536917077 | 0.999860533 | 0.226057063 | 0.999798691 | -0.72661161 |
| FBtr0079500 | Acp1 | 0.000202054 | 1.732372175 | 0.999860533 | 0.09758795 | 0.999798691 | -0.885490426 |
| FBtr0079607 | Mur29B | 3.32E-08 | 2.203753518 | 0.999860533 | 0.197413922 | 0.999798691 | -1.07312875 |
| FBtr0079758 | CG32984 | 0.045654093 | -3.439907422 | 0.999860533 | 0.645752982 | 0.999798691 | 0.552908824 |
| FBtr0080006 | CG5096 | 0.015470626 | 1.614508876 | 0.999860533 | -0.367414093 | 0.999798691 | -0.31965643 |
| FBtr0080334 | Phae1 | 0.011916271 | 3.572857584 | 0.999860533 | 0.065256589 | 0.999798691 | -0.816909518 |
| FBtr0080370 | Oatp33Eb | 0.001447556 | 3.186358438 | 0.999860533 | 0.103834241 | 0.999798691 | -0.605407439 |
| FBtr0080464 | CG16826 | 0.047411113 | -4.921706347 | 0.999860533 | 0.317931282 | 0.999798691 | -1.037449927 |
| FBtr0080755 | CG18480 | 0.021741609 | 2.935622985 | 0.999860533 | -0.630565562 | 0.999798691 | -0.742902444 |
| FBtr0081775 | CG18746 | 0.01108192 | 3.27852927 | 0.999860533 | -0.298709606 | 0.999798691 | -0.479629682 |
| FBtr0081777 | CG18745 | 0.006583154 | 3.550691932 | 0.999860533 | -0.050396783 | 0.999798691 | -1.293981626 |
| FBtr0081865 | CG11672 | 0.001634501 | 3.317037455 | 0.999860533 | -0.016395818 | 0.999798691 | -1.286000484 |
| FBtr0082900 | CG33109 | 0.002647488 | -1.460634359 | 0.999860533 | 0.451611841 | 0.999798691 | 0.152845503 |
| FBtr0083026 | CG3987 | 4.51E-05 | 1.512498647 | 0.999860533 | -0.106083913 | 0.999798691 | -0.319020279 |
| FBtr0083030 | Mf | 3.72E-05 | 1.206382413 | 0.999860533 | 0.099030162 | 0.999798691 | -0.484462628 |
| FBtr0083164 | CG5399 | 2.78E-06 | 3.575384524 | 0.999860533 | -0.231166289 | 0.999798691 | -1.022391377 |
| FBtr0083372 | CG8907 | 4.13E-08 | 1.300100455 | 0.999860533 | -0.074027186 | 0.999798691 | -0.587237507 |
| FBtr0083470 | AttD | 0.029084942 | 2.102904461 | 0.999860533 | -0.332978501 | 0.999798691 | -0.752156062 |
| FBtr0083763 | CG31221 | 0.999961787 | 0.737679608 | 0.000862597 | 1.050809868 | 0.830377559 | 0.554170152 |
| FBtr0083823 | CG4783 | 2.91E-05 | 2.545384042 | 0.999860533 | 0.095659943 | 0.999798691 | -1.191087191 |
| FBtr0084901 | CG5028 | NA | NA | NA | NA | 0.005199103 | 5.269815553 |
| FBtr0084957 | Kaz-m1 | 0.000550781 | 3.632660457 | 0.999860533 | 0.32125163 | 0.999798691 | -1.174018295 |
| FBtr0085305 | CG9988 | 0.019318853 | 2.186267427 | 0.999860533 | 0.356091476 | 0.999798691 | -1.435141947 |
| FBtr0085472 | CG7567 | 1.09E-06 | 3.047754006 | 0.999860533 | -0.16837695 | 0.999798691 | -1.418760398 |
| FBtr0086983 | Amy-d | 3.40E-09 | 2.577135928 | 0.999860533 | -0.291518954 | 0.999798691 | -0.70999857 |
| FBtr0087004 | Amy-p | 1.84E-12 | 4.110027459 | 0.999860533 | -1.017646884 | 0.999798691 | -1.522510028 |
| FBtr0087792 | CG13321 | 3.85E-05 | 3.747204916 | 0.999860533 | -0.096087692 | 0.999798691 | -0.77244778 |
| FBtr0087796 | CG13324 | 0.000164657 | 4.546214478 | 0.999860533 | -0.329679139 | 0.999798691 | -1.364380871 |
| FBtr0087797 | CG13323 | 4.51E-05 | 4.453581565 | 0.999860533 | -0.627626237 | 0.999798691 | -1.70978278 |
| FBtr0087854 | CG12374 | 0.012707045 | 4.556063987 | 0.999860533 | -1.246325839 | 0.999798691 | -1.934042089 |
| FBtr0088161 | alphaTry | 0.001040886 | 4.610885525 | 0.999860533 | -0.787534445 | 0.999798691 | -2.016153397 |
| FBtr0088432 | Def | 4.45E-06 | 1.378119365 | 0.999860533 | -0.3287707 | 0.999798691 | 0.025894016 |
| FBtr0088709 | PGRP-SC2 | 0.012076665 | 1.325852668 | 0.999860533 | -0.482132556 | 0.999798691 | -0.559767456 |
| FBtr0088778 | sut2 | 0.007779424 | 1.290837399 | 0.999860533 | -0.068761444 | 0.999798691 | -0.560034221 |
| FBtr0088798 | CG30371 | 0.031396721 | -1.970061931 | 0.999860533 | 0.603708922 | 0.999798691 | 0.629346054 |
| FBtr0088809 | CG8708 | 0.011739827 | -4.088796084 | 0.999860533 | -0.412198494 | 0.999798691 | 0.549657534 |
| FBtr0089084 | Eph | 0.999961787 | -0.3365599 | 0.999860533 | -1.156438859 | 9.25E-08 | -6.107714474 |
| FBtr0089323 | CG10725 | 0.010179506 | 2.858875766 | 0.999860533 | -0.981221046 | 0.999798691 | -1.668348149 |
| FBtr0089630 | CG10910 | 0.030325169 | 3.023928918 | 0.999860533 | -0.688787051 | 0.999798691 | -1.145744004 |
| FBtr0091498 | lectin-37Da | 0.043765258 | 2.443514123 | 0.999860533 | 0.061982808 | 0.999798691 | -0.489790991 |
| FBtr0100028 | obst-H | 0.000407687 | 2.801483865 | 0.999860533 | -0.536833144 | 0.999798691 | -0.344557865 |
| FBtr0100880 | mt:ND4L | 0.107949664 | -0.494579195 | 0.001724056 | -1.24427752 | 0.999798691 | -0.278179692 |
| FBtr0112405 | CG34212 | 0.005354159 | -4.088747384 | 0.999860533 | 0.335785619 | 0.999798691 | 0.288960446 |
| FBtr0112413 | CG34220 | 0.005518417 | 2.297703365 | 0.999860533 | 0.519683572 | 0.999798691 | -0.914126126 |
| FBtr0112526 | CG34324 | 0.002778253 | 2.696535128 | 0.999860533 | 0.167758038 | 0.999798691 | -1.247946825 |
| FBtr0114524 | Pgant4 | 0.004076224 | 2.997652431 | 0.999860533 | 0.148339914 | 0.999798691 | -1.341591531 |
| FBtr0290275 | tgy | 0.00374776 | 3.498024408 | 0.999860533 | 0.302355178 | 0.999798691 | -1.851606978 |
| FBtr0299517 | CG34452 | 0.023266727 | 1.909840845 | 0.999860533 | 0.16160219 | 0.999798691 | -0.851202763 |
| FBtr0300506 | CG42397 | 2.91E-05 | 4.470662763 | 0.999860533 | -0.681259525 | 0.999798691 | -1.350865859 |
| FBtr0300942 | AttB | 0.020989344 | 1.740897672 | 0.999860533 | 0.124711255 | 0.999798691 | -0.838004139 |
| FBtr0301011 | CG34236 | 0.012734734 | 3.362552013 | 0.999860533 | 0.031279231 | 0.999798691 | -1.262284501 |
| FBtr0301977 | CG31077 | 0.034615916 | 3.355964165 | 0.999860533 | 0.155923446 | 0.999798691 | -1.340605874 |
| FBtr0302527 | CG33346 | 1.25E-08 | 4.447797727 | 0.999860533 | -0.187933471 | 0.999798691 | -1.691273512 |
| FBtr0302854 | Phae2 | 0.001328434 | 3.958342039 | 0.999860533 | -0.252689587 | 0.999798691 | -1.035802492 |
| FBtr0303096 | Scsalpha | 0.999961787 | 0.189905375 | 1.35E-06 | 5.882649017 | 0.999798691 | 2.414431053 |
| FBtr0303251 | Wnt6 | 0.023799394 | -5.196394155 | 0.999860533 | -0.12512825 | 0.999798691 | 0.025491502 |
| FBtr0304571 | RyR | 0.999961787 | -1.85101192 | 0.999860533 | -0.597287554 | 0.046679175 | 5.239173713 |
| FBtr0305282 | CG43134 | 0.005158473 | 3.915432642 | 0.999860533 | 0.145741206 | 0.999798691 | -1.510418195 |
| FBtr0305795 | AttC | 0.033457074 | 1.384195623 | 0.999860533 | -0.299003684 | 0.999798691 | -0.944843868 |
| FBtr0306289 | CG43236 | 0.000133305 | 1.148856362 | 0.999860533 | 0.031384637 | 0.999798691 | -0.21047466 |
| FBtr0306805 | CG9626 | 0.003698463 | -4.667810078 | 0.999860533 | 1.890598637 | 0.999798691 | -0.297857151 |
| FBtr0308229 | CG13810 | 7.37E-05 | 2.80924618 | 0.999860533 | 0.164078158 | 0.999798691 | -1.10885918 |
| FBtr0310341 | CrebB | 0.027771971 | -3.418573945 | 0.999860533 | -0.552829814 | 0.999798691 | 1.321894379 |
| FBtr0310431 | CG32633 | 8.70E-05 | 3.026437806 | 0.999860533 | -0.008520226 | 0.999798691 | -0.861819136 |
| FBtr0310455 | CG43680 | 1.48E-05 | -4.62766656 | NA | NA | 0.999798691 | 1.513355137 |
| FBtr0310456 | CG43679 | 0.00996018 | -3.515517529 | 0.999860533 | -0.400829925 | 0.999798691 | 0.066828361 |
| FBtr0331166 | Cht10 | 0.03801919 | 4.892066465 | 0.999860533 | -3.931794266 | 0.999798691 | -1.420313878 |
| FBtr0332131 | CG42255 | 1.25E-32 | 1.320266165 | 0.999860533 | -0.078147577 | 0.999798691 | -0.555492014 |
| FBtr0332542 | CG11672 | 0.007372266 | 3.850526101 | 0.999860533 | 0.309855336 | 0.999798691 | -1.933994734 |
| FBtr0334027 | CG5399 | 0.012734734 | 3.391333412 | 0.999860533 | -0.416508492 | 0.999798691 | -0.878315691 |
| FBtr0334150 | CG44013 | 0.012076665 | 3.708603551 | 0.999860533 | -0.791453925 | 0.999798691 | -1.720434868 |
| FBtr0335017 | asRNA:CR44106 | 0.018318226 | 2.211271247 | 0.999860533 | 0.128355578 | 0.999798691 | -0.996007681 |
| FBtr0335461 | CG44142 | 0.02139924 | -3.641530183 | 0.999860533 | 0.405541014 | 0.999798691 | 0.545995649 |
| FBtr0339105 | CG44250 | 0.049420238 | 3.316292967 | 0.999860533 | 0.754095068 | 0.999798691 | -0.323609367 |
| FBtr0339640 | CG31358 | 0.023151529 | 3.16956332 | 0.999860533 | -0.172608788 | 0.999798691 | -1.532047025 |
| FBtr0340272 | lncRNA:CR44404 | 0.049426755 | 1.673257002 | 0.999860533 | -0.380719359 | 0.999798691 | -0.491514423 |
| FBtr0343047 | CG15043 | 0.005354159 | 3.478596326 | 0.999860533 | -0.498781853 | 0.999798691 | -1.119059409 |
| FBtr0344451 | CG45080 | 0.006874185 | -4.44362139 | 0.999860533 | 0.286700302 | 0.999798691 | -0.449033169 |
| FBtr0345521 | Amy-p | 4.80E-05 | 5.396352897 | 0.999860533 | -1.042837629 | 0.999798691 | -1.945550431 |
| FBtr0346383 | whe | 0.001932977 | -5.350102624 | 0.999860533 | 1.177034201 | 0.999798691 | 1.051610189 |
