# Supplementary material for: Transgenerational inheritance of ethanol preference is caused by maternal NPF repression
Source: eLife. 2019 Jul 9;8:e45391. doi: 10.7554/eLife.45391 (PMC6615861; doi:10.7554/eLife.45391)
Supplement: Supplementary file 5. [file elife-45391-supp5.docx]

**Supplementary file 5**. Drosophila stock list and source information.

| **name** | **genotype** | **source** | **stock number** |
| --- | --- | --- | --- |
| CS | + | Bosco Lab |  |
| OreR | + | Bosco Lab |  |
| Orb2[deltaQ] | Orb2[deltaQ] | Bosco Lab |  |
| NPF-Gal4 | y[1] w[*]; P{w[+mC]=NPF-GAL4.1}2 | Bloomington stock center | 25681 |
| UAS-NPF | UAS-NPF | Shen Lab |  |
| Elav-Gal4 | Elav-Gal4 | Bosco Lab |  |
| UAS-NPF[RNAi] | y[1] v[1]; P{y[+t7.7] v[+t1.8]=TRiP.JF02555}attP2 | Bloomington stock center | 27237 |
| UAS-NPFR[RNAi] | y[1] v[1]; P{y[+t7.7] v[+t1.8]=TRiP.JF01959}attP2 | Bloomington stock center | 25939 |
| UAS-Dcp1[RNAi] | y[1] v[1]; P{y[+t7.7] v[+t1.8]=TRiP.HM05120}attP2 | Bloomington Stock Center | 28909 |
| UAS-Drice[RNAi] | y[1] sc[*] v[1]; P{y[+t7.7] v[+t1.8]=TRiP.HMS00398}attP2 | Bloomington Stock Center | 32403 |
| Matα-Gal4 | w[*]; P{w[+mC]=matalpha4-GAL-VP16}V37 | Bloomington Stock Center | 7063 |
| ninaB[1] | w[*]/Dp(1;Y)y[+]; ninaB[1], P{w[+mC]=UAS-ninaB.G}3 | Bloomington Stock Center | 24776 |
| compound ch-II | C(2)EN, b[1] pr[1] | Bloomington stock center | 1112 |
| compound ch-II | C(2)EN, bw[1] sp[1] | Bloomington stock center | 1020 |
| compound ch-III | C(3)EN, Diap1[1] st[1] | Bloomington stock center | 1114 |
| compound ch-III | C(3)EN, st[1] cu[1] e[s] | Bloomington stock center | 1117 |
| Df(3)10642 | w[1118]; Df(3R)ED10642, P{3'.RS5+3.3'}ED10642/TM6C, cu[1] Sb[1] | Bloomington stock center | 9482 |
| Df(3)BSC472 | w[1118]; Df(3R)BSC472/TM6C, Sb[1] cu[1] | Bloomington stock center | 24976 |
| Df(3)BSC510 | w[1118]; Df(3R)BSC510/TM6C, Sb[1] cu[1] | Bloomington stock center | 25014 |
| yw | y[1]w[1] | Bloomington stock center | 1495 |
| amn[1] | amn[1] | Bloomington stock center | 5954 |
| MB-Gal4(switch) | P{MB-Switch} | Roman Lab |  |
| Orb2[RNAi] | y[1] v[1]; P{TRiP.JF02376}attP2 | Bloomington stock center | 27050 |
